# Effects of Maternal Age on Fetus and Perinatal Outcomes in a Tertiary Care Center: An Observational Study

**DOI:** 10.31729/jnma.8834

**Published:** 2024-12-31

**Authors:** Sonam Chaudhary, Narayan Mahotra, Pooja Paudyal

**Affiliations:** 1 Department of Clinical Physiology, Maharajgunj Medical Campus, Maharajgunj, Kathmandu, Nepal; 2 Department of Gynaecology and Obstetrics, Maharajgunj Medical Campus, Maharajgunj, Kathmandu, Nepal

**Keywords:** *maternal age*, *Nepalese*, *perinatal outcome*

## Abstract

**Introduction:**

The early and late pregnancy both can be hazardous for mother and child. The study aims to explore the maternal age group among Nepalese women who delivered in Tribhuvan University Teaching Hospital and its effects on perinatal outcomes.

**Methods:**

An observational cross-section study was carried out from February to September, 2023 with the records of maternity cases at a tertiary care hospital after receiving ethical approval from the Institutional Review Committee [Reference number: 130(6-11) E2-2 079/080]. The records of maternity cases of four years duration from April, 2018 onwards were included. Data was entered in Microsoft Excel 2016 and descriptive analysis was done.

**Results:**

Out of 13062 maternity cases, the prevalence of pregnancy in normal reproductive age was 12035 (92.13%). A total of 882 (6.67%) maternity cases were in advanced age and 145 (1.11%) were in teen age. The alive newborns which were more in normal reproductive age was 12008 (99.77%) and stillbirth which was more in teen age pregnancy was 4 (2.75%). The low-birth-weight newborns distributed more in teen age pregnancy was 44 (30.34%) and high birth weight newborns distributed more in advanced age pregnancy was 18 (2.04%).

**Conclusions:**

The abnormal birth weight and stillbirths were common in teen age and advanced age pregnancy.

## INTRODUCTION

A safe motherhood is dependent upon maternal age which is between 20-30 years.^1,2^ However, nearly 11% of births worldwide are to women between 1519 years, seen mostly in low and middle income countries.^3^ In Nepal, 17% of adolescent women are already mothers or pregnant with their first child,^4^ increasing the risks of preterm birth, low birth weight and neonatal mortality.^5,6^ Similarly, advanced maternal age which is 35 or more years is associated with declined fertility and increased risk of complications like caesarean delivery, preterm birth and perinatal death.^7-11^ The prevalence of advance maternal age was 12.3%, ranging from 2.8% in Nepal to 31.1% in Japan.^12^ This shows that the maternal age range vary widely in our community. Thus, this study aims to find the distribution of maternal age and its effect on perinatal outcomes in hospital settings. The result will generate an evidence-based information to aware the women adequately about the pregnancy outcomes at different maternal ages which can affect their procreation choices.

## METHODS

This was a hospital based retrospective observational cross-section study carried out in Tribhuvan University Teaching Hospital, Maharajgunj, Kathmandu, Nepal. The study was carried out from February, 2023 AD after getting the ethical clearance from the Institutional Review Committe of Institute of Medicine [Reference number: 130(6-11) E2 079/80]. The records of all the maternity cases from April, 2018 to March, 2022 were taken into consideration. The records of maternity cases of any age and who delivered baby normally or via caesarean section were included from the hospital records by total sampling. Any maternity cases of non- Nepali origin or cases with missing information and unclear records were excluded from the study. The hospital being a tertiary care centre at Kathmandu, Nepal, the cases are referred from in and outside of Kathmandu valley and so the records utilized as data in this study can be generalized.

The independent variable considered in the study was maternal age, and dependent variables were fetal birth weight, still birth frequencies and the mode of deliveries opted by the mothers. The maternal age group was again divided into: 1. Teen age pregnancy, 2. Normal reproductive age pregnancy, 3. Advance age pregnancy. According to World Health Organization (WHO), teenage pregnancy means pregnancy between 10 and 19 years of age.^13^ The advanced age pregnancy is considered if maternal age is 35 or more years at the time of delivery, whereas very advanced maternal age is defined as 40 or more at the time of delivery.^8^ The maternal age in between these two groups were considered of normal reproductive age group. The maternal age group in this study was categorized with this consideration. The mothers delivering only one newborn of any fetal viability were considered as singleton pregnancy, two newborns were considered as twin pregnancy and 3 newborns were considered as triplet pregnancy.

In the fetal outcomes, the birth weight of the newborns was taken from the hospital records. The newborns with birth weight lower than 2.5 kg were considered as low birth weight and more than 4 kg was considered as macrosomia. The newborns having birth weight 2.5 kg to 4 kg were considered normal.^14^

For fetal viability, the fetal status was considered alive if the fetus was born alive and dead if no viability. These dead fetus after 28 weeks of pregnancy was considered as stillbirth.^15^ The mode of deliveries recorded during data collection was vaginal deliveries and cesarean deliveries. The normal deliveries with different degree of tear or episiotomies or vacuum assisted deliveries were all included under vaginal deliveries.

Data was entered in Microsoft Excel 2016. The statistical analysis was done with using SPSS Statistics for Windows, version 16.0 (SPSS Inc., Chicago, Ill., USA) Descriptive statistics were used to describe and summarize the data. Categorical variables were analyzed as percentage, and continuous variable with normal distribution were presented as mean±SD.

## RESULTS

A total of 13062 maternity cases were included in this retrospective study. The maternal age ranged between 15-47 years of age with mean age of 27.20±4.49 years. The pregnancy belonging to normal reproductive age was 12035 (92.13%) and that to advanced age was 882 (6.75%) and 145 (1.11%) belonged to teen age. There were 12940 (99.06%) singleton pregnancy, 6792 (52%) vaginal delivery, 13008 (98.65%) alive newborn and 10931 (82.90%) newborn had normal birth weight (Table 1).

**Table 1:** General descriptives of the cases recorded (n=13062).

Variables		Frequency n(%)
Age	Teen age pregnancy	145 (1.11)
	Normal age pregnancy	12035 (92.13)
	Advance age pregnancy	882 (6.75)
Pregnancy Types	Singleton pregnancy	12940 (99.06)
	Twin pregnancy	121 (0.92)
	Triplet pregnancy	1(0.02)
Mode of delivery	Vaginal delivery	6792 (51.99)
	Caesarean delivery	6270 (48.01)
Newborn variables (13185)		
Viability	Alive	13008 (98.65)
	Dead	177 (1.34)
Birth weight	High birth weight	121 (0.91)
	Normal birth weight	10931 (82.90)
	Low birth weight	2133 (16.17)

There were 12008 (98.76%) live newborn in normal reproductive age pregnancy, 862 (97.73%) in advance age and 138 (95.17%) in teen age pregnancy (Table 2).

**Table 2:** Distribution of newborn viability among different maternal age groups (n=13062).

Maternal age group	Newborn Viability		
	Alive n(%)	Still birth n(%)	Dead n(%)
Teen age (145)	138 (95.17)	4 (2.75)	3 (2.06)
Normal Reproductive age (12035)^*^	12008 (98.76)	97 (0.79)	53 (0.43)
Advanced age (882)	862 (97.73)	11 (1.24)	9 (1.02)

* Newborn in normal reproductive age pregnancy = 12158

The newborn having normal birth weight were 10164 (83.59%) in the group with normal reproductive age pregnancy, 668 (75.73%) in advanced age and 99 (68.27%) in teenage pregnancy (Table 3).

**Table 3:** Distribution of newborns (by birth weight) among different maternal age groups (n = 13062).

	Newborns		
Maternal age group	Low birth weight n(%)	Normal birth weight n(%)	High birth weight n(%)
Teen age (145)	44 (30.34)	99 (68.27)	2 (1.37)
Normal reproductive age (12035)^*^	1893 (15.12)	10164 (83.59)	101 (0.83)
Advanced age (882)	196 (22.22)	668 (75.73)	18(2.04)

* Newborn in normal reproductive age pregnancy = 12158

There were 6243 (51.87%) vaginal deliveries in normal reproductive age, 341(38.66%) in advance age and 105 (72.41%) in teenage pregnancy (Table 4).

**Table 4:** Distribution of maternal age group among the various methods of delivery opted (n=13062).

Maternal age group	Vaginal deliveries n(%)	Caesarean Section deliveries n(%)
Teen age (145)	105 (72.41)	40 (27.58)
Normal reproductive age (12035)	6243 (51.87)	5792 (48.12)
Advance age (882)	341 (38.66)	541 (61.33)

## DISCUSSION

This retrospective study was designed to find the effects of maternal age distribution in fetal outcomes in Nepalese settings. This study has taken maternal age, number of fetus, viability of fetus, birth weight of newborn and method of delivery opted in considerations for meeting the objectives.

The maternal age distribution in the data recorded was between 15 years and 47 years with mean age 27.20±4.49 years. The percentage of teen-age pregnancy was 1.11% in this study. This finding doesn't match with the reports of Demographic and Health Survey, 2016 which found the prevalence of teenage pregnancy was 17% with maximum (22%) in rural settings and 13% in urban areas.^4^ A systematic review and meta-analysis had also reported 13.2% prevalence of teen-age pregnancy.^16^ These reports have also claimed that the teenage pregnancy reduces with the education level. The education and awareness can also be credited for the maternity cases to come to the hospital for the deliveries which were recorded in this study. This could be a potential factor for such decrease prevalence of teenage pregnancy in this retrospective study of hospital setting. This study has found the distribution of vaginal delivery more in teen age pregnancy (72.41%) which is supported by other studies in Nepal and other countries as well.^16,17^ However, a high rate of caesarean delivery was also seen in teen age pregnancy in a retrospective study conducted in Romanian hospital.^18^

This study has also found that the distribution of low-birth-weight babies was 30.34% and still birth distribution was 2.75% in teen age pregnancy. Similar increase incidence of low birth weight and still birth among teen age pregnancy is supported by other studies in India and Canada as well. ^19,20^ The low birth weight among teen age pregnancy was comparatively more 19.4%, and the distribution of stillbirth was 1.7% among this maternity age in a systemic review previously done in Nepal.^16^

The percentage distribution of advanced age pregnancy was 6.75% in this study. A study conducted in Manipal Teaching Hospital, Pokhara, Nepal between 2019-2020 has also reported 5.73% advanced age pregnancy.^11^ A previous study had revealed the prevalence of advanced maternal age was 12.3% in 29 different countries considered and it was 2.8% in Nepal.^12^ This shows the increasing trend of advanced age pregnancy in Nepal. The factors related with education and career could be potential reason for increase in advance age pregnancy in modern women which need further validations. The caesarean sections were mostly opted by the advanced age pregnancy (61.33%) in this study which is in consistent with studies done earlier.^11^ The prevalence of low birth weight in advanced maternal age was 22.22% in this study. A similar study conducted previously in Nepal which reported the prevalence of low birth weight among the advance age pregnancy was 8.65%.^11^ The percentage wise distribution of macrosomia was more in advanced age pregnancy in this study which is parallel to other studies as well.^21,22^ The increase in birth weight of newborns could be related with the preference of caesarean deliveries among the advanced age pregnancy in the study population. There are many literatures to support that the advanced maternal age is a risk factor for many complications in pregnancy, including low birth weight or macrosomia of neonates.^1,23-26^

The prevalence of twin and triplet pregnancy was 0.92% and 0.0075% in this retrospective study. A previous study about the twins in Nepal had reported 1.61% twin and 0.91% triplet pregnancy in Nepal.^27^ Similarly, a retrospective study of Eastern Nepal in 2014 had reported 0.92% twin pregnancy.^28^

This retrospective study has thus analyzed the maternity cases of nearly 4 years in a tertiary care hospital, Nepal. The finding of this study has showed the percentage distribution of adverse perinatal outcomes being more among teen age pregnancy and advanced age pregnancy which can relate with the fact that normal reproductive age group is associated with better perinatal outcomes. The data doesn't represent the maternity cases which occur in settings outside hospital and thus the results could pay few disparities in comparison to the mass surveys done in local settings. Similar studies done in different regions of Nepal can further generate more impactful results to address the importance of maternal age for safe motherhood outcomes. Similarly, the records utilized in this study can include data from the same mother having more than one delivery during the study period. The study period also coincides with the COVID- era which can affect the prevalence of maternity cases or the perinatal outcomes as well in this hospital settings. The lack of normal sample size distribution in various categories of variables considered lead to lack of application of inferential statistics in the data generated which if done could have provided with more impactful insight about the effects of maternal age in perinatal outcomes. Since safe motherhood program is conducted in national level, it is important to conduct similar researches in future for the safety of mother and the child.

## CONCLUSIONS

The study has concluded that most of the pregnant women visiting hospital belonged to normal reproductive age group and had better perinatal outcomes. The adverse perinatal outcomes like viability of the fetus and their abnormal birth weight were common in teen age or advanced age pregnancy.
